# Co‐treatment with the seed of *Carthamus tinctorius* L. and the aerial part of *Taraxacum coreanum* synergistically suppresses Aβ_25–35_‐induced neurotoxicity by altering APP processing

**DOI:** 10.1002/fsn3.3768

**Published:** 2023-10-18

**Authors:** Mei Tong He, Ji Hyun Kim, Eun Ju Cho

**Affiliations:** ^1^ College of Korean Medicine Gachon University Seongnam Korea; ^2^ Department of Food Science and Nutrition Gyeongsang National University Jinju Korea; ^3^ Department of Food Science and Nutrition & Kimchi Research Institute Pusan National University Busan Korea

**Keywords:** amyloidogenic pathway, apoptosis, *Carthamus tinctorius* L. seed, synergy effect, *Taraxacum coreanum*

## Abstract

Accumulation of β‐amyloid peptide (Aβ) induces neurotoxicity, which is the primary risk factor in the pathogenesis of Alzheimer's disease (AD). The cleavage of amyloid precursor protein (APP) by the β‐ (BACE) and γ‐ (PS1, PS2) secretases is a critical step in the amyloidogenic pathway. The induction of neuronal apoptosis by Aβ involves increased expression of B‐cell lymphoma protein 2 (Bcl‐2)‐associated X (Bax) and decreased Bcl‐2 expression. The seed of *Carthamus tinctorius* L. (CTS) and the aerial part of *Taraxacum coreanum* (TC) are traditional herbs used to treat several neurodegenerative diseases. In this study, the neuroprotective effects of co‐treatment with CTS and TC on Aβ‐induced neurotoxicity in SH‐SY5Y neuroblastoma cells and the underlying mechanisms were investigated. CTS, TC, and the co‐treatment (CTS + TC) were added to Aβ_25–35_‐treated SH‐SY5Y cells. CTS + TC synergistically increased cell viability and inhibited reactive oxygen species production. CTS + TC resulted in significant downregulation of BACE, PS1, PS2, and APP, as well as the 99‐aa C‐terminal domain of APP, compared with either CTS or TC alone. Compared with the single herbs, co‐treatment with CTS and TC markedly decreased the expression of Bax and increased the expression of Bcl‐2, consistent with its anti‐apoptotic effects. These findings suggest that co‐treatment with CTS and TC may be useful for AD prevention.

## Graphical abstract

Co‐treatment with *Carthamus tinctorius* L. seed and *Taraxacum coreanum* synergistically protect neuronal cell damage compared to the single herb. The *C. tinctorius* L. seed and *T. coreanum* mixture inhibits the generation of β‐amyloid and decreases reactive oxygen species‐mediated apoptosis.

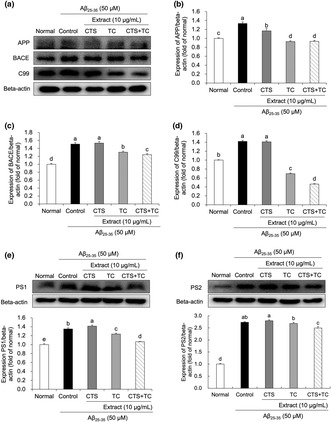

## 1 INTRODUCTION

The prevalence and associated morbidity of Alzheimer's disease (AD) are growing rapidly owing to the increase in life expectancy worldwide. Several studies have demonstrated that AD neuropathogenesis is triggered by the accumulation of β‐amyloid peptide (Aβ) produced by the cleavage and processing of amyloid precursor protein (APP), in a process called the amyloidogenic pathway (Prasansuklab & Tencomnao, 2013; Selkoe & Hardy, 2016). APP is a transmembrane protein expressed at high levels in the brain (Thinakaran & Koo, 2008). In the amyloidogenic pathway, APP is first cleaved by β‐secretase (BACE), generating a soluble N‐terminal fragment of APP and a membrane‐bound, 99‐residue C‐terminal fragment (C99). C99 is then cleaved by γ‐secretase (presenilin 1, PS1; presenilin 2, PS2) to produce Aβ (Zhang et al., 2011).

The neurotoxicity mediated by Aβ accumulation regulates multiple mechanisms, including oxidative stress (Huang et al., 1999), neuronal apoptosis (Paradis et al., 1996), and inflammation (Giovannini et al., 2002). Therefore, the development of therapeutics targeting this pathway is the subject of intensive research (Zhou et al., 2018). Aβ_25–35_ is reportedly more neurotoxic than the active fragment of full‐length Aβ, due to its C‐terminal methionine (Butterfield & Sultana, 2011; Stepanichev et al., 2005). In addition, Aβ_25–35_ has been shown to be toxic to cultured neuronal cells (Hertel et al., 1997; Shanmugam & Polavarapu, 2004), thereby providing a model system for neurodegenerative diseases.

*Carthamus tinctorius* L., or safflower, belongs to the family *Asteraceae*. It was originally cultivated in the Middle East, India, and Africa (Weiss, 1971). Traditionally, its seeds have been used for the treatment of cardiovascular and bone diseases (Bae et al., 2002; Koyama et al., 2006). Studies have demonstrated the antioxidative (Yu et al., 2014), anti‐acetylcholinesterase (Peng et al., 2017), and cognition‐improving (Choi et al., 2018) effects of *C. tinctorius* L. seed. Another member of the *Asteraceae*, the white dandelion *Taraxacum coreanum*, is native to South Korea (Lee et al., 2013). *T. coreanum* has been reported to exert anti‐inflammatory (Lee et al., 2013), neuroprotective (Yoon et al., 2017), anti‐cancer (Yamabe et al., 2014), and antioxidative (Lee & Oh, 2015) effects. Many pairs of drugs exert synergistic effects (Wagner & Ulrich‐Merzenich, 2009). A previous study reported synergistic effects of the *C. tinctorius* L. seed and *T. coreanum* combined at a 5:5 ratio in Aβ_25–35_‐induced cognitive impairment in vivo (He et al., 2020). However, the neuroprotective effect of co‐treatment with the seed of *C. tinctorius* L. and the aerial part of *T. coreanum* on neuronal cells is still unclear. Therefore, we aimed to identify synergistic neuroprotective effects of the seed of *C. tinctorius* L. and the aerial part of *T. coreanum* in Aβ_25–35_‐treated SH‐SY5Y neuroblastoma cells by measuring viability, reactive oxygen species (ROS) levels, and lactate dehydrogenase (LDH) release. We also analyzed the mechanisms underlying amyloidogenesis and apoptosis.

## 2 MATERIALS AND METHODS

### 2.1 Reagents

Cell‐culture reagents, including Dulbecco's Modified Eagle's Medium (DMEM), fetal bovine serum (FBS), penicillin–streptomycin solution, and trypsin–EDTA solution, were purchased from Welgene Inc. 3‐(4,5‐Dimethylthiazol‐2‐yl)‐2,3‐diphenyl tetrazolium bromide (MTT) was purchased from Bio Basic Inc. Aβ_25–35_ (≥97% HPLC) and 2′,7′‐dichlorofluorescein diacetate (DCF‐DA) were obtained from Sigma‐Aldrich. Dimethyl sulfoxide (DMSO) was purchased from Daejung Chemicals & Metals Co., Ltd. Primary and secondary antibodies were purchased from Sigma‐Aldrich and Cell Signaling Technology, Inc.

### 2.2 Plant materials and sample preparation

Water extracts of the seed of *C. tinctorius* L. (CTS), the aerial part of *T. coreanum* (TC), and their 1:1 mixture (CTS + TC) were obtained from the National Institute of Horticultural and Herbal Science, Rural Development Administration (Eumseong, Korea). In brief, the seed of *C. tinctorius* L. and the aerial part of *T. coreanum* were collected, dried, and pulverized. For this study, water extraction (90°C, 8 h) was used to obtain plant samples, which were filtered through 200‐mesh filter paper. Samples were vacuum concentrated at 60°C and freeze‐dried (Kim et al., 2020). The yields of CTS, TC, and their mixture were 8.3%, 30.6%, and 26.3%, respectively. The samples were dissolved in DMSO before use.

### 2.3 Aβ_25–35_ stocks

Aβ_25–35_ was dissolved in sterilized distilled water to obtain a stock concentration of 1 mM and incubated at 37°C for 72 h to allow aggregation. The stock solution was diluted in DMEM to a final concentration of 50 μM prior to use.

### 2.4 Cell culture and treatment

SH‐SY5Y neuroblastoma cells were purchased from ATCC (Manassas, VA, USA) and cultured in DMEM supplemented with 10% FBS and 1% penicillin–streptomycin at 37°C in a 5% CO_2_ humidified atmosphere. The experimental conditions were as follows: (1) culture medium only (normal), (2) culture medium with Aβ_25–35_ (control), (3) the seed of *C. tinctorius* L. (CTS, 10 μg/mL) + Aβ_25–35_, (4) the aerial part of *T. coreanum* (TC, 10 μg/mL) + Aβ_25–35_, and (5) co‐treatment with the seed of *C. tinctorius* L. and the aerial part of *T. coreanum* at a ratio of 1:1 (CTS + TC, 10 μg/mL) + Aβ_25–35_.

### 2.5 MTT assay

Cell viability was measured using the MTT assay (Mosmann, 1983). Briefly, cells (2.5 × 10^5^ cells/well) were seeded in 96‐well plates and cultured for 24 h. CTS, TC, and CTS + TC at 10 μg/mL were pretreated for 4 h, followed by 50 μM Aβ_25–35_ for all conditions except the normal group, and incubated for another 24 h. Cells were incubated with MTT solution at 37°C for 4 h, and formazan crystals were dissolved in DMSO. Absorbance was noted at 540 nm using a microplate reader (Rayto Life and Analytical Sciences Co., Ltd.).

### 2.6 DCF‐DA assay

ROS production was measured using the fluorescent probe DCF‐DA. Briefly, cells were seeded in a 96‐well black plate for 24 h and pretreated with CTS, TC, and CTS + TC for 4 h, followed by Aβ_25–35_ treatment. Cells were then incubated with 80 μM DCF‐DA for 30 min. Fluorescence was read at an excitation wavelength of 480 nm and an emission wavelength of 535 nm using the FLUO star OPTIMA (BMG Labtech).

### 2.7 LDH release assay

LDH level was measured using a Cytotoxicity Detection Kit (TaKaRa Bio Inc., #MK401). Cells were seeded in a 96‐well plate for 24 h and pretreated with CTS, TC, and CTS + TC for 4 h, followed by Aβ_25–35_ treatment, and incubated for 24 h. After incubation, the supernatant was assayed for LDH levels according to the manufacturer's instructions. Briefly, 100 μL of supernatant was mixed with the LDH reaction mixture in an equal amount and incubated at room temperature in the dark for 30 min. Absorbance was read at 490 nm using the microplate reader (Rayto Life and Analytical Sciences Co., Ltd.).

### 2.8 Western blotting

Cells were lysed in RIPA buffer containing a 1× protease inhibitor cocktail. Protein concentrations were measured using a protein assay kit (Bio‐Rad) according to the manufacturer's instructions. Samples containing equal amounts of protein (15 μg per lane) were resolved on 10% or 13% SDS gels and transferred to methanol‐activated polyvinylidene difluoride membranes. Nonspecific binding was blocked for 1 h with PBST containing 5% non‐fat milk. Subsequently, the membranes were incubated overnight at 4°C with primary antibodies (anti‐APP/C99, A8717, 1:1000; anti‐BACE, #5606, 1:1000; anti‐PS1, #5643, 1:1000; anti‐PS2, #9979, 1:1000; anti‐Bax, #2772, 1:1000; and anti‐Bcl‐2, ab196495, 1:500). Membranes were washed three times for 10 min each with PBST and then incubated with secondary antibodies (anti‐rabbit IgG, HRP, #7074, 1:1000). Following three washes with PBST, immunoreactivity was detected using a chemiluminescent detection system (Davinch Chemi™).

### 2.9 Statistical analysis

Data are presented as mean ± S.D. Statistical analysis was performed using one‐way analysis of variance (ANOVA) followed by Duncan's multiple test (SPSS, version 23.0); statistical significance was set at *p* < .05.

## 3 RESULTS

### 3.1 Co‐treatment with the seed of *C. tinctorius* L. and the aerial part of *T. coreanum* synergistically attenuates Aβ_25–35_‐induced neurotoxicity

**FIGURE 1:**
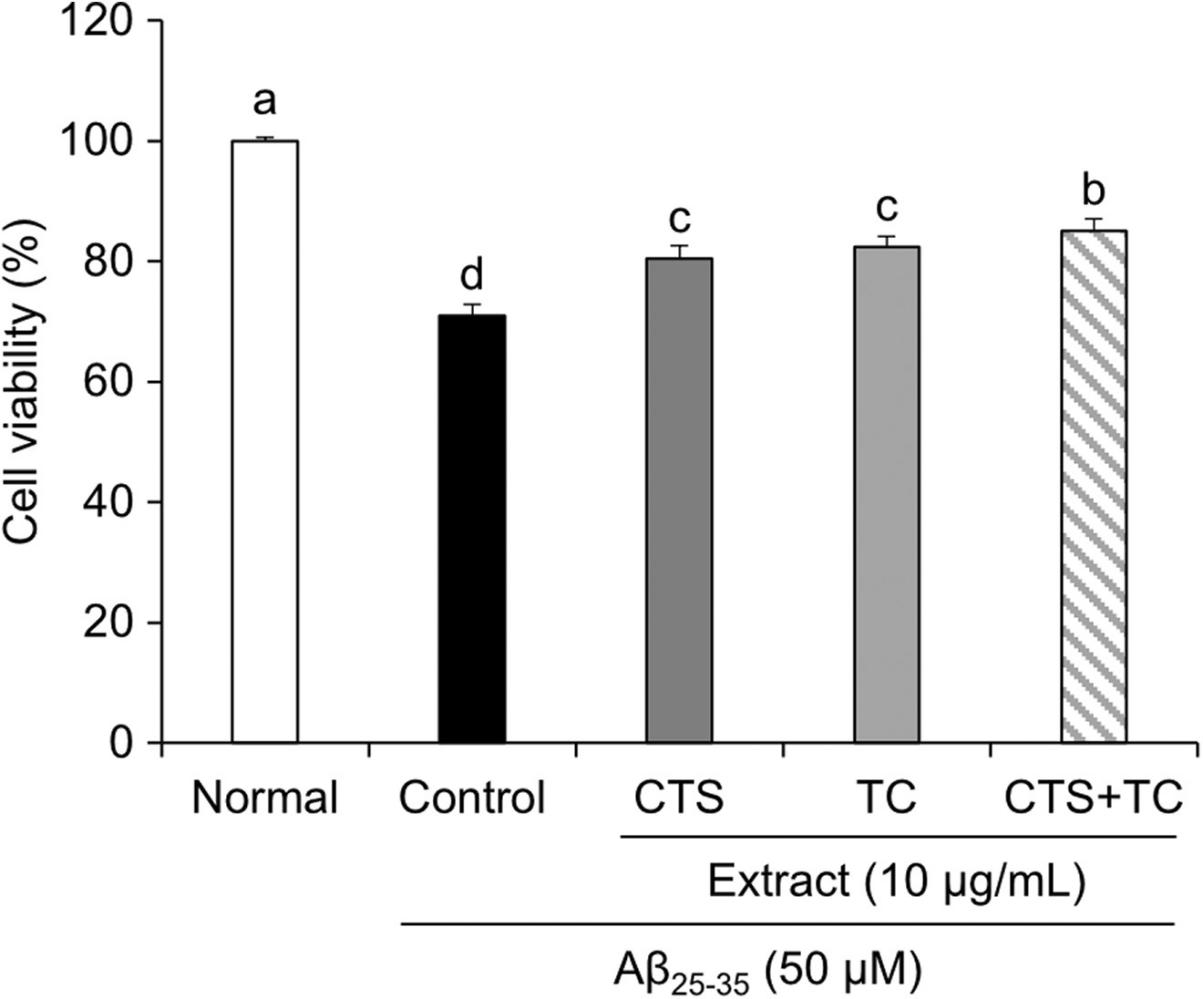
Viability in SH‐SY5Y cells treated with Aβ_25–35_. Values are means ± SD. ^a–d^Means with different letters are significantly different (*p* < .05) by Duncan's multiple range test. CTS, the seed of *C. tinctorius* L.; CTS + TC, co‐treatment with the seed of *C. tinctorius* L. and the aerial part of *T. coreanum*; TC, the aerial part of *T. coreanum*.

To test for co‐treatment with CTS and TC synergy, an MTT assay was conducted. As shown in Figure 1, control cells treated with only Aβ_25–35_ exhibited a 30% increase in cell death compared with the normal cells. In contrast, the CTS, TC, and CTS + TC conditions showed significant increases in viability compared with the Aβ_25–35_ control. The cell viability of CTS + TC increased higher than that of CTS or TC alone, showing 85% of the viability.

### 3.2 Co‐treatment with the seed of *C. tinctorius* L. and the aerial part of *T. coreanum* synergistically attenuates Aβ_25–35_‐induced ROS production

**FIGURE 2:**
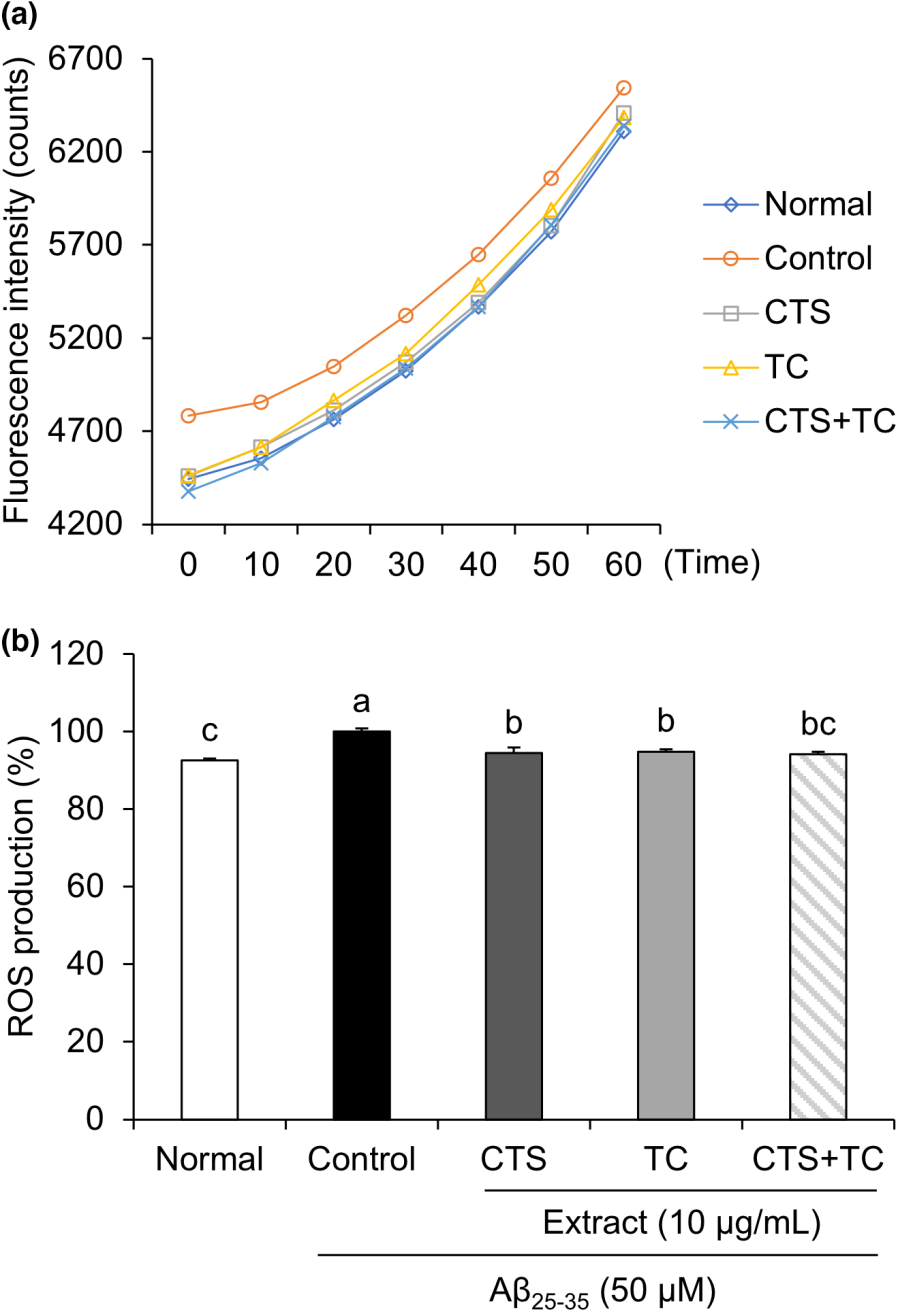
Reactive oxygen species in SH‐SY5Y cells treated with Aβ_25–35_. Fluorescence intensity during 60 min (a) and ROS production at 60 min (b). Values are means ± SD. ^a–c^Means with different letters are significantly different (*p* < .05) by Duncan's multiple range test. CTS, the seed of *C. tinctorius* L.; CTS + TC, co‐treatment with the seed of *C. tinctorius* L. and the aerial part of *T. coreanum*; TC, the aerial part of *T. coreanum*.

As shown in Figure 2, ROS levels were increased in Aβ_25–35_‐treated control cells compared with those in normal cells. However, ROS levels decreased in both CTS and TC treatment conditions compared to control cells. Moreover, CTS + TC significantly suppressed ROS production induced by Aβ_25–35_ treatment.

### 3.3 Co‐treatment with the seed of *C. tinctorius* L. and the aerial part of *T. coreanum* synergistically attenuates Aβ_25–35_‐induced LDH release

**FIGURE 3:**
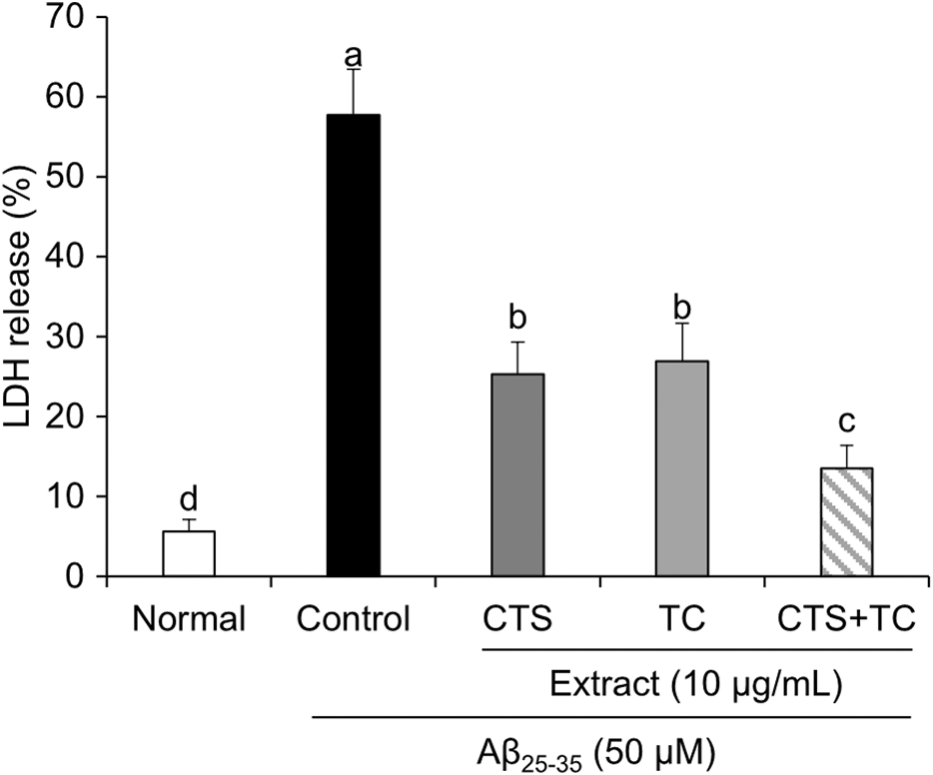
Lactate dehydrogenase release in SH‐SY5Y cells treated with Aβ_25–35_. Values are means ± SD. ^a–d^Means with different letters are significantly different (*p* < .05) by Duncan's multiple range test. CTS, the seed of *C. tinctorius* L.; CTS + TC, co‐treatment with the seed of *C. tinctorius* L. and the aerial part of *T. coreanum*; TC, the aerial part of *T. coreanum*.

To further investigate the protective effect of CTS and TC against cell damage, LDH release was measured. LDH release markedly increased after exposure to Aβ_25–35_ (Figure 3). However, LDH release was significantly decreased by CTS and TC, especially in the group of co‐treatment with CTS and TC, whose LDH level was lower than that of the CTS or TC group.

### 3.4 Co‐treatment with the seed of *C. tinctorius* L. and the aerial part of *T. coreanum* synergistically exerts an anti‐amyloidogenic effect

**FIGURE 4:**
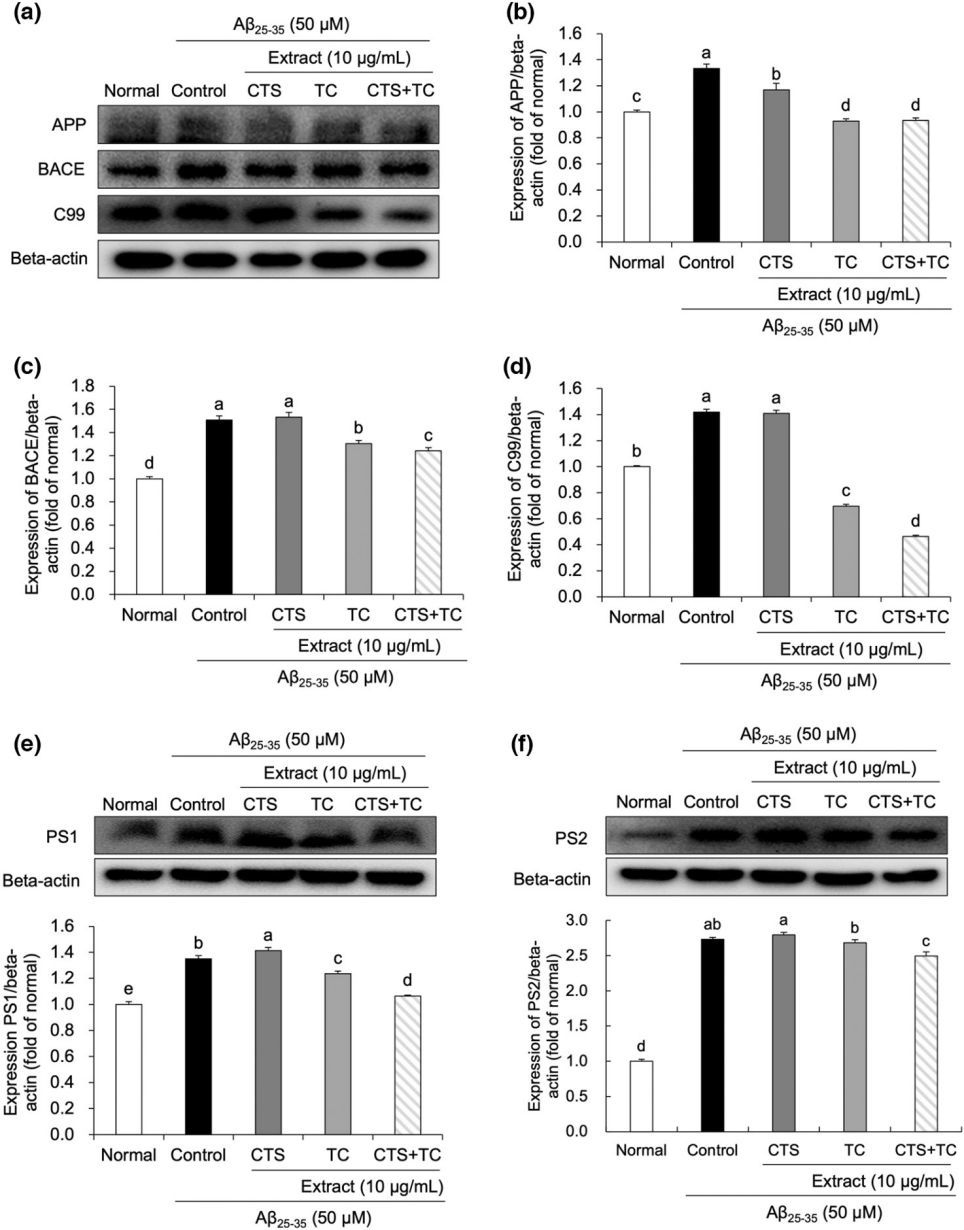
Expression of amyloidogenic‐related proteins in Aβ_25–35_‐treated SH‐SY5Y cells. Protein expression (a) and statistical analysis of APP (b), BACE (c), C99 (d), PS1 (e), and PS2 (f). Values are means ± SD. ^a–e^Means with different letters are significantly different (*p* < .05) by Duncan's multiple range test. CTS, the seed of *C. tinctorius* L.; CTS + TC, co‐treatment with the seed of *C. tinctorius* L. and the aerial part of *T. coreanum*; TC, the aerial part of *T. coreanum*.

We examined protein expression using Western blotting (Figure 4). The Aβ_25–35_ treatment significantly induced amyloidogenesis‐related protein expressions in the control cells compared to the normal cells. The result showed that co‐treatment with CTS and TC suppressed APP cleavage. Moreover, BACE expression was decreased by CTS + TC more effectively than CTS or TC alone. The release of C99 was lower following co‐treatment with CTS and TC than CTS or TC alone treatment. Moreover, PS1 and PS2 protein expression after CTS + TC treatment was significantly reduced compared to that after CTS or TC treatment.

### 3.5 Co‐treatment with the seed of *C. tinctorius* L. and the aerial part of *T. coreanum* synergistically exerts an anti‐apoptotic effect

**FIGURE 5:**
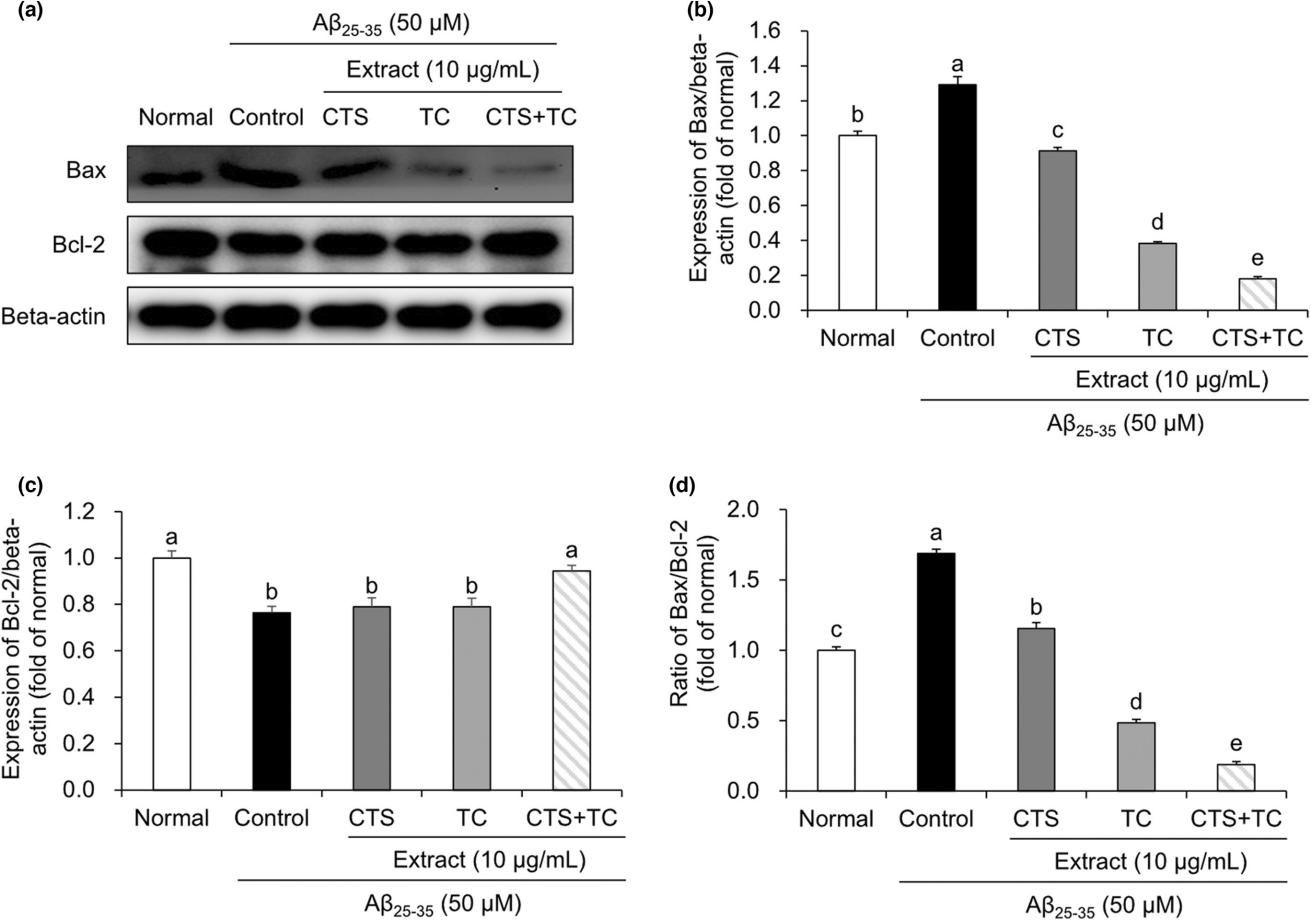
Expression of Bax and Bcl‐2 in Aβ_25–35_‐treated SH‐SY5Y cells. Protein expression and statistical analysis of Bax and Bcl‐2 (a–c), and ratio of Bax/Bcl‐2 (d). Values are means ± SD. ^a–e^Means with different letters are significantly different (*p* < .05) by Duncan's multiple range test. CTS, the seed of *C. tinctorius* L.; CTS + TC, co‐treatment with the seed of *C. tinctorius* L. and the aerial part of *T. coreanum*; TC, the aerial part of *T. coreanum*.

As shown in Figure 5, the expression of Bax protein increased in Aβ_25–35_‐treated control cells compared to that in normal cells; it was attenuated by CTS and TC treatment. Moreover, CTS + TC synergistically decreased Bax levels to a greater extent than did CTS or TC. Aβ_25–35_‐treated control cells showed significantly reduced Bcl‐2 protein expression compared to the normal cells. CTS‐ or TC‐treated cells were not significantly different from control cells; however, CTS + TC treatment increased Bcl‐2 levels, suggesting that CTS + TC synergistically protects against Aβ_25–35_‐induced apoptosis. In addition, in Aβ_25–35_‐treated cells, the ratio of Bax to Bcl‐2 was significantly increased than in control cells. However, co‐treatment with CTS and TC decreased the Bax/Bcl‐2 ratio compared to treatment with CTS or TC.

## 4 DISCUSSION

Herbal therapy has been widely used for the prevention and treatment of AD. For example, *Ginkgo biloba* clinically alleviates cognitive disorders in elderly individuals (Snitz et al., 2009). However, AD has a complex pathogenesis with multiple processes participating in neuroinflammation (Cai et al., 2014), oxidative stress (Zhu et al., 2007), and apoptosis (Shimohama, 2000). Both CTS and TC have been reported to exert neuroprotective effects. CTS inhibits acetylcholinesterase activity in scopolamine‐induced cognitive impairment (Kim et al., 2019). TC positively regulates HO‐1/Nrf‐2 signaling in mouse hippocampal HT22 cells under neurotoxic conditions (Yoon et al., 2017). Our previous in vivo study showed that combining CTS and TC synergistically attenuated Aβ‐induced neurotoxicity via altering APP processing (He et al., 2020). However, the protective effects of herbal combinations against Aβ_25–35_‐induced neuronal cell damage remained to be elucidated. Therefore, the present study investigated the synergistic beneficial effects of the seed of *C. tinctorius* L. and the aerial part of *T. coreanum* on Aβ_25–35_‐induced neurotoxicity. Martínez and Pascual (2007) reported that Aβ_25–35_‐treated SH‐SY5Y cells significantly upregulate the expression of genes involved in the c‐fos and MAPK signaling cascades associated with neurotoxicity. Thus, SH‐SY5Y cells treated with Aβ_25–35_ present a suitable in vitro model for AD.

The amyloidogenic pathway processes mutant APP proteins and eventually produces Aβ via β‐ and γ‐secretase‐mediated protein cleavage (Chow et al., 2010). Cleavage of APP by β‐secretase generates an Aβ‐contained C99 fragment, which has been reported to have toxic effects, leading to neuronal death (Castro et al., 2019; Pulina et al., 2019; Ribe et al., 2008). In this study, the levels of APP and BACE, as well as C99, increased after the cells were exposed to Aβ_25–35_, and they were recovered by CTS and TC treatment. Notably, CTS + TC synergistically decreased the levels of BACE and C99, suggesting that co‐treatment of CTS and TC might have an inhibitory effect on the generation of C99 from β‐secretases. PS1 and PS2 encode γ‐secretase, which releases Aβ from C99 (Bolduc et al., 2016; Li et al., 2000). The protein expressions of PS1 and PS2 increased in the control cells; in contrast, CTS + TC significantly decreased the expression of both. These results suggest that co‐treatment with the seed of *C. tinctorius* L. and the aerial part of *T. coreanum* effectively and synergistically inhibits Aβ generation.

ROS can be produced in the mitochondria, plasma membrane, and endoplasmic reticulum, resulting in oxidative stress and ultimately cell apoptosis (Ma et al., 2017). A previous study reported apoptosis induction in Aβ_25–35_‐treated SH‐SY5Y cells (Li et al., 1996). As apoptosis plays an important role in several neurodegenerative diseases, its regulation is closely related to the maintenance of the normal cell cycle (Huang et al., 2019). The Bcl‐2 family members have played roles in the regulation of the apoptotic pathway (Ghate et al., 2014). In apoptosis, Bcl‐2 prevents Bax from releasing cytochrome *c*, thereby exerting an anti‐apoptotic effect. In contrast, Bax is a pro‐apoptotic factor that facilitates the release of cytochrome *c* to promote apoptosis (Kulsoom et al., 2018). Additionally, alteration of the Bax/Bcl‐2 ratio has been reported as an important factor in apoptosis (Jiang et al., 2014). A low Bax/Bcl‐2 ratio caused by downregulation of Bax and upregulation of Bcl‐2 has been demonstrated to resist apoptosis (Zhu et al., 2015). In this study, the expression of the apoptosis‐related proteins Bax and Bcl‐2 was examined. In Aβ_25–35_‐treated cells, the ratio of Bax to Bcl‐2 was significantly increased compared with control cells. However, CTS + TC decreased the Bax/Bcl‐2 ratio compared to treatment with CTS or TC alone. This suggests that the synergistic protective effect of co‐treatment with the seed of *C. tinctorius* L. and the aerial part of *T. coreanum* against Aβ_25–35_‐induced neurotoxicity is related to the regulation of the neuronal apoptotic pathway.

## 5 CONCLUSION

Taken together, the co‐treatment with the seed of *C. tinctorius* L. and the aerial part of *T. coreanum* protected against cell damage and inhibited ROS production in Aβ_25–35_‐treated SH‐SY5Y cells, as well as attenuated Aβ_25–35_‐induced neurotoxicity by downregulating the expression of proteins related to amyloidogenesis and decreasing the Bax/Bcl‐2 ratio. These findings suggest that co‐treatment with the seed of *C. tinctorius* L. and the aerial part of *T. coreanum* synergistically protects Aβ_25–35_‐treated neuronal cells from neurotoxicity. Our results provide a model system for studying the neuroprotective effects of the seed of *C. tinctorius* L. and the aerial part of *T. coreanum*. However, multiple mechanisms underlying the effects of this herbal pair remain to be elucidated to identify its functions in AD prevention and treatment.

## AUTHOR CONTRIBUTIONS

**Mei Tong He:** Conceptualization (equal); data curation (equal); formal analysis (equal); investigation (equal); methodology (equal); software (equal); visualization (equal); writing – original draft (equal). **Ji Hyun Kim:** Formal analysis (equal); investigation (equal); writing – review and editing (equal). **Eun Ju Cho:** Conceptualization (equal); methodology (equal); resources (equal); supervision (equal); writing – review and editing (equal).

## CONFLICT OF INTEREST STATEMENT

The authors declare that they do not have any conflicts of interest.

## ETHICS STATEMENT

This study does not involve any human or animal testing.

## INFORMED CONSENT

Written informed consent was obtained from all study participants.

## Data Availability

The data associated with this research are available and can be obtained by contacting the corresponding author.
